# Screening of Crude Plant Extracts with Anti-Obesity Activity

**DOI:** 10.3390/ijms13021710

**Published:** 2012-02-06

**Authors:** Changhyun Roh, Uhee Jung

**Affiliations:** Radiation Research Division for Biotechnology, Advanced Radiation Technology Institute (ARTI), Korea Atomic Energy Research Institute (KAERI), 1266, Shinjeong-dong, Jeongeup, Jeonbuk 580-185, Korea

**Keywords:** anti-obesity, plant extracts, screening, lipid inhibition

## Abstract

Obesity is a global health problem. It is also known to be a risk factor for the development of metabolic disorders, type 2 diabetes, systemic hypertension, cardiovascular disease, dyslipidemia, and atherosclerosis. In this study, we screened crude extracts from 400 plants to test their anti-obesity activity using porcine pancreatic lipase assay (PPL; triacylglycerol lipase, EC 3.1.1.3) *in vitro* activity. Among the 400 plants species examined, 44 extracts from plants, showed high anti-lipase activity using 2,4-dinitrophenylbutyrate as a substrate in porcine pancreatic lipase assay. Furthermore, 44 plant extracts were investigated for their inhibition of lipid accumulation in 3T3-L1 cells. Among these 44 extracts examined, crude extracts from 4 natural plant species were active. *Salicis Radicis Cortex* had the highest fat inhibitory activity, whereas *Rubi Fructus*, *Corni Fructus*, and *Geranium nepalense* exhibited fat inhibitory capacity higher than 30% at 100 μg/mL in 3T3-L1 adipocytes, suggesting anti-obesity activity. These results suggest that four potent plant extracts might be of therapeutic interest with respect to the treatment of obesity.

## 1. Introduction

Obesity is a chronic metabolic disorder caused by an imbalance between energy intake and expenditure. Overweight and obesity are defined as abnormal or excessive fat accumulation that presents a risk to health [1–4]. Many scientific communities have become increasingly interested in the molecular regulation of triglyceride synthesis and in pharmaceutical approaches to reduce fat absorption and storage due to phytochemicals, presenting an exciting opportunity for the discovery of newer anti-obesity agents [5–7]. The regulation of fatty acid and triglyceride availability in biological responses depends on the activity of lipolytic enzymes present in fatty acid metabolism in adipose tissue [8–10].

The characterization and identification of several genes involved in lipid metabolism have yielded a rich pool of potential targets for drugs to treat obesity and other metabolic syndromes [11–14]. Pancreatic lipase, the main lipid digesting enzyme, removes fatty acids from the α and α′ positions of dietary triglycerides, which yield the lipolytic product β-monoglyceride and long chain saturated and polyunsaturated fatty acids. Inhibition of pancreatic lipase is an attractive targeted approach for the discovery of potent anti-obesity agents for obesity treatment [15,16].

One of the screening strategies used in the discovery of anti-obesity drugs is to search for potent lipase inhibitors from plant extracts. Plants have been used as traditional natural medicines for healing many diseases. In particular, various oriental medicinal plants are reported to have biological activity [17]. In this study, we screened crude extracts from natural sources as potential anti-obesity agents by monitoring their anti-lipase activity. We also elucidated anti-obesity effects on lipid accumulation in cultured 3T3-L1 adipocytes by measuring Oil Red O staining and triglyceride (TG) contents as indicators of lipid accumulation. Among the plant extracts screened, the four most promising extracts (*Rubi Fructus*, *Corni Fructus*, *Salicis Radicis Cortex*, and *Geranium nepalense*) might be of therapeutic interest with respect to the treatment of obesity. To the best of our knowledge, these plant extracts have not been previously screened for their lipid inhibitory activity.

## 2. Experimental Methods

### 2.1. Chemicals

Four-hundred kinds of plants were purchased from a plant extract bank at Korea Research Institute of Bioscience & Biotechnology (KRIBB), and were authenticated by H.K. Lee. A collection of voucher specimens is available for confirmation in the Plant Extract Bank, Korea Research Institute of Bioscience and Biotechnology, Daejeon, Republic of Korea. The plant extracts were dissolved in dimethylsulfoxide (DMSO) and used as samples for screening tests. Orlistat, *p*-nitrophenyl butyrate (NPB), and lipase (Type II: from Porcine pancreas) were purchased from Sigma-Aldrich Chemical Co. (St. Louis, MO, USA). All reagents were of the highest grade available.

### 2.2. Preparation of Natural Extracts

The plants were extracted three times with ethanol, and extracts were obtained through the removal of the solvent during evaporation. The concentrated samples were stored at −20 °C for further study. Extracts were dissolved in DMSO at a final concentration that did not affect enzyme activity within the total volume (1%).

### 2.3. Pancreatic Lipase Inhibition Assay

Porcine pancreatic lipase (PPL, type II) activity was measured using *p*-nitrophenyl butyrate (*p*-NPB) as a substrate. The method used for measuring the pancreatic lipase activity was modified from that previously described by Kim, *et al.* and Zheng, *et al.* [18,19]. PPL stock solutions (1 mg/mL) were prepared in a 0.1 mM potassium phosphate buffer (pH 6.0) and the solutions were stored at −20 °C. To determine the lipase inhibitory activity, the extracts (final concentrations 100, 50, 25, 10, 5, 2.5, 1.25 μg/mL) or Orlistat (at same concentrations) as a positive control were pre-incubated with PPL for 1 h in a potassium phosphate buffer (0.1 mM, pH 7.2, 0.1% Tween 80) at 30 °C before assaying the PPL activity. The reaction was then started by adding 0.1 μL NPB as a substrate, all in a final volume of 100 μL. After incubation at 30 °C for 5 min, the amount of *p*-nitrophenol released in the reaction was measured at 405 nm using a UV-Visible spectrophotometer (BioTek Synergy HT, Winooski, VT, USA). The activity of the negative control was also examined with and without an inhibitor. The inhibitory activity (I) was calculated according to the following formula:

Inhibitory activity (I%)=100-((B-b)/(A-a)×100)

where A is the activity without inhibitor; a is the negative control without inhibitor; B is the activity with inhibitor; and b is the negative control with inhibitor. DMSO was used as negative control and its activity was also examined.

### 2.4. Cell Culture and Differentiation

3T3-L1 preadipocytes were obtained from ATCC (Manassas, VA, USA). 3T3-L1 preadipocytes were grown in DMEM supplemented with 10% (v/v) heat-inactivated FBS at 37 °C in an atmosphere containing 5% CO_2_. To induce adipocyte differentiation, 2-day post-confluent 3T3-L1 preadipocytes (day 0) were stimulated for 48 h (day 2) with an inducer (10 μg/mL insulin, 2.5 μM dexamethasone, and 0.5 mM 3-isobutyl-1-methylxanthine) including natural extracts, and then maintained for 6 days (day 8) in DMEM supplemented with 10% FBS and 10 μg/mL insulin including natural extracts. 3T3-L1 cells were treated with natural extracts in DMEM supplemented with 10% FBS for 2 days (day 10). To examine the effect of natural extracts on adipocyte differentiation in 3T3-L1 cells, the media and natural extracts were changed every 2 days until the end of the experiment at day 10.

### 2.5. Cell Viability and Oil Red O Staining Intracellular Triglycerides

Cell viability was determined colorimetrically using an MTT assay [20]. Cells cultured in DMEM medium were treated with natural extracts at a final concentration of 100 μg/mL for 2 days, and then incubated with a 5 mg/mL MTT (3-(4,5-dimetyl-2-thiazolyl)-2,5-diphenyltetrazoliumbromide) solution (Sigma) for 3 h. After the cells were dissolved in 0.04 N HCl (in isopropanol), the formazane level was analyzed by measuring the optical density (OD) at 570 nm (against OD at 630 nm) [18]. 3T3-L1 adipocytes were washed with PBS and fixed with 10% formalin for 30 min. After two washes with distilled water, the cells were stained for at least 1 h at room temperature in a freshly diluted Oil Red O solution (Oil Red O stock solution used is 0.5% Oil Red O in isopropanol). Finally, the dye retained in the 3T3-L1 cells was eluted with isopropanol and quantified by measuring the absorbance at 500 nm.

### 2.6. Measurement of Triglyceride (TG) and Glycerol

Cellular TG contents were measured using a commercial TG assay kit (Asan Pharm. Co., Seoul, Republic of Korea) according to the manufacturer’s instructions. Cells were treated with plant extracts at concentrations of 100 μg/mL in 6-well plates during adipocyte differentiation for 6 days. The cells were washed twice with phosphate-buffered saline (PBS), scraped in 75 μL of a homogenizing solution (154 mM KCl, 1 mM EDTA and 50 mM Tris, pH 7.4), and sonicated to homogenize cell suspension. The residual cell lysate was centrifuged at 3000× g for 5 min at 25 °C to remove fat layers. The supernatants were assayed for TG and protein contents. TG was normalized to the protein concentration determined by the Bradford assay using bovine serum albumin (BSA) as standard. The results were expressed in milligrams of TG per milligram of cellular protein. Lipolysis was assessed through the measurement of glycerol released (Free glycerol reagent, Sigma, St. Louis, MO, USA) into the medium, according to the manufacturer’s instructions [21]. Briefly, differentiated 3T3-L1 adipocytes were treated with plant extracts for 24 h. After incubation, 50 μL of the medium was incubated with 200 μL of a Free Glycerol Reagent for 15 min at room temperature. The glycerol was quantified by measuring absorbance at 540 nm.

### 2.7. Statistics

Statistical analysis was done by using one way analysis of variance using the SPSS program [22]. Values of *p* < 0.05 were considered to be statistically significant.

## 3. Results and Discussion

### 3.1. Anti-Lipase Activity of Crude Natural Source Extracts

**Figure 1:**
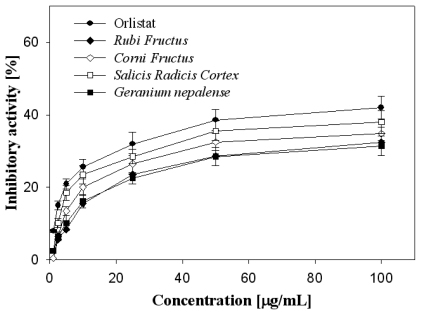
Porcine pancreatic lipase (PPL) inhibitory activities of *Rubi Fructus*, *Corni Fructus*, *Salicis Radicis Cortex*, *Geranium nepalense*. Orlistat was used as a positive control. Experiments have been performed in triplicate.

Four-hundred crude extracts were prepared from natural plant species found in Korea or Asia and their anti-lipase activity was investigated at a concentration of 100 μg/mL for PPL inhibition. The inhibitory activities towards pancreatic lipase are reported in Table 1. Among the 400 plant extracts examined, 44 crude extracts from natural plant species, at a concentration of 100 μg/mL, significantly inhibited PPL *in vitro* activity when using a 2,4-dinitrophenylbutirate-based assay. Among those examined, four of the extracts showed a relatively high anti-lipase activity of more than 30%. The significant inhibition of PPL was observed up to 32.5% with *Rubi Fructus* fruit, 34.8% with *Corni Fructus* fruit, 38% with *Salicis Radicis Cortex* bark and 31.4% with *Geranium nepalense* whole grass, respectively. Treatment with Orlistat (at final concentration 100 μg/mL) as a positive control, a well-known anti-lipase agent, significantly inhibited the PPL activity up to 42%. Orlistat, a hydrogenated derivative of lipstatin, is the only pancreatic lipase inhibitor currently approved for a long-term treatment of obesity. Crude extracts of *Rubi Fructus*, *Corni Fructus*, *Salicis Radicis Cortex*, and *Geranium nepalense* were further investigated for their PPL inhibitory effects at different concentrations, and a dose-response curve was obtained, as shown in Figure 1.

### 3.2. Effect of Cell Viability and Lipid Inhibition in 3T3-L1 Cells

**Figure 2:**
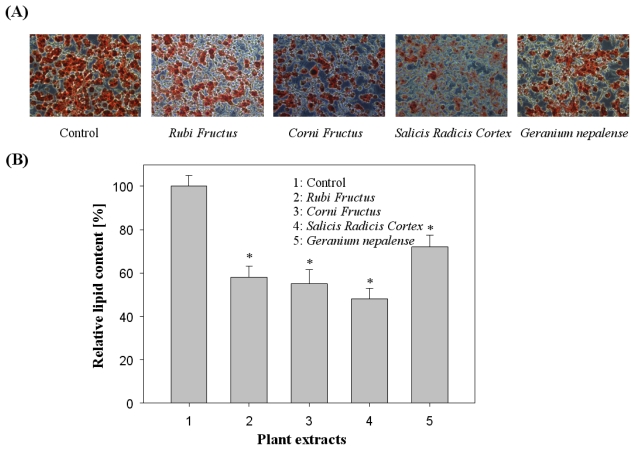
Effects of plant extracts on Oil Red O staining in cultured 3T3-L1 adipocytes. (**A**) Effects of plant extracts on fat droplet formation in 3T3-L1 cells. It was stained with Oil Red O dye and examined using a light microscope; (**B**) Relative lipid content by quantification method of Oil Red O staining. Data are presented as average ± SD (*n* = 3). * indicates *p* < 0.05.

The 3T3-L1 adipocytes were cultured and differentiated in a Dulbecco Modified Eagle Medium containing 10% fetal bovine serum for 6 to 8 days in the absence and presence of 44 plant extracts (at a final concentration, 100 μg/mL) according to differentiating protocols. Extracts were dissolved in DMSO at a final concentration that did not affect cell activity within the total volume (1%). As shown in Figure 2, the 44 plant extracts at 100 μg/mL attenuated lipid accumulation in differentiated adipocytes as evidenced by Oil Red O staining. Among the natural extracts examined, the four potent natural extracts (*Rubi Fructus*, *Corni Fructus*, *Salicis Radicis Cortex*, and *Geranium nepalense*) were found to significantly reduce lipid accumulation in 3T3-L1 adipocytes, suggesting anti-obesity activity. In Figure 2A,B, the effects of plant extracts on fat droplet formation in 3T3-L1 cells, and inhibition through the quantification method of Oil Red O staining, were presented. To examine the effect of plant extracts on cell viability of 3T3-L1 preadipocytes, we performed an MTT assay, which assesses cell viability by measuring mitochondrial activity in 3T3-L1 cells treated with concentrations of crude extracts. The potent crude extracts (*Rubi Fructus*, *Corni Fructus*, *Salicis Radicis Cortex*, and *Geranium nepalense*) screened have relatively low cytotoxicity to 3T3-L1 preadipocyte cells. The cell cytotoxicity remained approximately 90–100% (data not shown).

**Figure 3:**
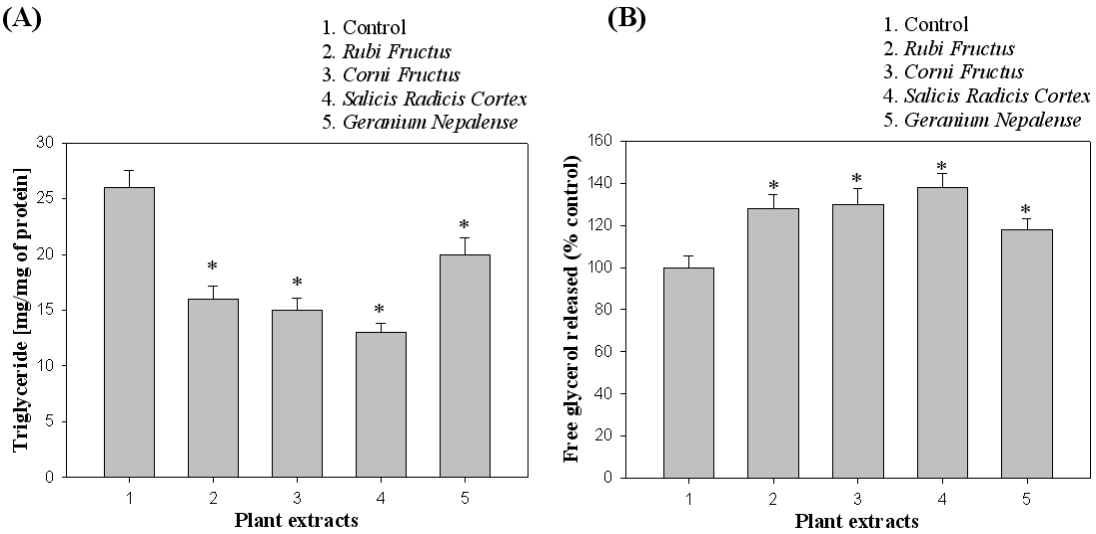
Effects of plant extracts on adipocyte differentiation in 3T3-L1 cells. (**A**) Triglyceride (TG) accumulation was measured by TG contents of 3T3-L1 cells differentiated in the presence of natural extracts; (**B**) Lipolysis was assessed by the measurement of glycerol released into the medium. Data are presented as average ± SD (*n* = 3). * indicates *p* < 0.05.

As shown in Figure 3A, lipid accumulation was measured based on the TG contents of 3T3-L1 cells differentiated in the presence of natural extracts. Furthermore, the lipolysis was assessed through the measurement of glycerol released in culture medium for 24 h incubation, as shown in Figure 3B. The four natural extracts that exhibited inhibitory activity towards pancreatic lipase (*Rubi Fructus*, *Corni Fructus*, *Salicis Radicis Cortex*, and *Geranium nepalense*) were found to inhibit triglyceride accumulation in 3T3-L1 adipocytes and trigger lipid metabolism process to glycerol release.

*Rubi Fructus*, the unripe fruit of *Rubus Chingii Hu* belonging to the Rosaceae family, can be found in many parts of the Asia, especially in China. In general, *Rubi Fructus* has estrogenic effect, promote lymphocyte proliferation and elevate testosterone level by promoting the activity of steroid synthesizing enzymes and by inhibiting their degradation [23]. *Corni Fructus*, the pulp of *Cornus officinalis sieb.* belonging to the Cornaceae in family, can be found in China, Japan and Korea. The biological activity of *Corni Fructus* was reported to relieve cyclophosphamid-induced leukopenia and to have antibacterial effects [24]. *Salicis Radicis Cortex* belonging to the Ulmaceae in family was reported to have antioxidant, antitumor, antimetastatic effects [25], to increase NO synthase activity [26], to lower LDL cholesterol levels [27], to prevent cancer development and metastasis [28] and to be effective in wound healing, angiogenesis, and cardiovascular disease [29]. *Geranium Nepalense* belonging to the Geraniaceae in family was reported to have antibacterial and antifungal effects [30]. Even though these four plants have reported to have various biological activities, there was no report indicating them to have lipid inhibitory activity.

## 4. Conclusions

Obesity is a risk factor for metabolic syndromes, and a flexible approach for the treatment of obesity is to promote early adipogenesis in adipose tissue, thereby leading to the replacement of enlarged adipocytes that secrete inflammatory factors with small adipocytes. In this study, we screened crude anti-obesity drugs from four-hundred plant extracts on *in vitro* enzymatic lipase activity. Among 400 plant extracts examined, 44 extracts from plant extracts significantly inhibited against *in vitro* anti-lipase activity. Among 44 natural extracts examined, the four plant extracts were active to inhibit lipid formation in 3T3-L1 adipocytes, suggesting their use as crude anti-obesity agents. Among them, *Salicis Radicis Cortex* had highest fat inhibition activity. Therefore, these results suggest that these four active plant extracts could be useful for prevention or treatment of obesity.

## Figures and Tables

**Table 1:** Forty four plant extracts that inhibit porcine pancreatic lipase (PPL).

No.	Latin Name	Scientific Name	Family Name	Plant Part	Inhibition (%) *
1	*Platycodi Radix*	*Platycodon grandiflorum* A. De Candolle	Campanulaceae	Root	3.5 ± 0.7
2	*Aconiti Tuber*	*Aconitum carmichaeli* Debeaux	Ranunculaceae	Root	12.1 ± 1.1
3	*Cannabis Semen*	*Cannabis sativa* Linne	Moraceae	Seed	7.7 ± 0.2
4	*Chaenomelis Fructus*	*Chaenomeles sinensis* (Thouin) Koehne	Rosaceae	Fruit	7.1 ± 0.7
5	*Actinidiae Fructus*	*Actinidia chinensis*	Actinidiaceae	Fruit	3.2 ± 0.2
6	*Tribuli Semen*	*Tribulus terrestris*	Zygophyllaceae	Seed	2.5 ± 0.6
7	*Lilie Bulbus*	*Lilium brownii* var*. viridulun Baker*	Liliaceae	Stem	6.5 ± 1.2
8	*Luffae Fructus Retinervus*	*Luffa cylindrica* Roemer	Cucurbitaceae	Fruit	8.2 ± 1.4
9	*Crataegi Fructus*	*Crataegus pinnatifida Bunge* var*. typica Schneider*	Rosaceae	Fruit	5.5 ± 1.2
10	*Puerariae Radix*	*Pueraria thunbergiana* Bentham	Leguminosae	Root	3.2 ± 0.1
11	*Nardostachyos Rhizoma*	*Nardostachys chinensis* Batal	Valerianaceae	Rhizoma	6.4 ± 0.5
12	*Zizyphi Fructus*	*Zizyphus jujuba Miller* var*. inermis Rehder*	Rhamnaceae	Fruit	6.6 ± 1.5
13	*Akebiae Caulis*	*Akebia quinata* Decaisne	Lardizabalaceae	Stem	11.5 ± 0.1
14	*Quisqalis Fructus*	*Quisqualis indica* Linné	Combretaceae	Fruit	11.8 ± 0.3
15	*Loranthi Ramulus*	*Loranthus parasticus* Merr.	Loranthaceae	Whole grass	13.5 ± 0.7
16	*Schizandrae Fructus*	*Schizandra chinensis* Baillon	Schizandraceae	Fruit	5.8 ± 0.2
17	*Lonicerae Folium*	*Lonicera japonica* Thunberg	Caprifoliaceae	Stem	6.4 ± 0.5
18	*Rehmaniae Radix Preparata*	*Rehmannia glutinosa*	Scrophulariaceae	Root	6.8 ± 1.1
19	*Dipsaci Radix*	*Dipsacus asperoides* C. Y. Cheng et T. M. Ai	Dipsacaceae	Root	12.5 ± 0.3
20	*Morindae Radix*	*Morinda officinalis* How	Rubiaceae	Root	3.9 ± 0.7
21	*Perillae Semen*	*Perilla sikokiana* Nakai	Labiatae	Seed	8.4 ± 0.3
22	*Pruni Nakaii Semen*	*Prunus nakaii* Leveille	Rosaceae	Seed	7.7 ± 0.6
23	*Meliae Fructus*	*Melia azedarach Linné* var*. japonica Makino*	Meliaceae	Fruit	8.6 ± 0.8
24	*Bletillae Rhizoma*	*Bletilla striata* (Thunberg) Reichenbach fil.	Orchidaceae	Rhizoma	12.1 ± 0.4
25	*Hedyotidis Diffusae Herba*	*Oldenlandia diffusa* (Willd.) Roxburgh	Rubiaceae	Whole grass	1.80 ± 0.4
26	*Hoelen rubra*	*Poria cocos wolf*	Polyporaceae	Bark	12.7 ± 0.5
27	*Gastrodiae Rhizoma*	*Gastrodia Blume*	Orochidaceae	Rhizoma	8.4 ± 0.8
28	*Gentianae Scabrae Radix*	*Gentiana scabra*	Sympetalae	Root	7.5 ± 1.1
29	*Cuscutae Semen crudus*	*Cuscuta chinensis* Lamark	Convolvulaceae	Seed	5.8 ± 0.4
30	*Tetrapanacis Medulla*	*Tetrapanax papyriferus* K. Koch	Araliaceae	Stem	6.6 ± 0.7
31	*Fritillariae Bulbus*	*Fritillaria thunbergii* Miquel	Liliaceae	Stem	8.7 ± 0.5
32	*Patriniae Radix*	*Patrinia villosa* Jussieu	Valerianaceae	Root	7.7 ± 1.4
33	*Scutellariae Radix*	*Scutellaria baicalenis* Georgi	Labiatae	Root	8.7 ± 0.4
34	*Astragali Radix*	*Astragalus membranaceus* Bunge	Leguminosae	Root	7.4 ± 1.4
35	*Phellodendri Cortex*	*Phellodendron amurense* Ruprecht	Rutaceae	Bark	11.5 ± 0.2
36	*Drynariae Rhizoma*	*Drynaria fortunei* Smith.	Polypodiaceae	Rhizoma	10.5 ± 0.4
37	*Rubi Fructus*	*Rubus coreanus* Miquel	Rosaceae	Fruit	32.5 ± 1.1
38	*Eriobotriae Folium*	*Eriobotrya japonica* Lindley	Rosaceae	Leaf	9.8 ± 0.3
39	*Corni Fructus*	*Cornus officinalis* Siebold et Zuccarini	Cornaceae	Fruit	34.8 ± 2.3
40	*Forsythiae Fructus*	*Forsythia koreana* Nakai	Oleaceae	Fruit	5.7 ± 1.2
41	*Salicis Radicis Cortex*	*Ulmus darvidian* for*. Suberose*	Ulmaceae	Bark	38.0 ± 1.9
42	*Ammomi Tsao-ko Frucuts*	*Amomum tsao-ko* Crevost et Lemaire	Zingiberaceae	Fruit	7.5 ± 0.6
43	*Polygoni Avicularis Herba*	*Polygonum aviculare* Linné	Polygonaceae	Whole grass	21.2 ± 1.7
44	*Geranium Nepalense*	*Geranium thunbergii* Siebold et Zuccarini	Geraniaceae	Whole grass	31.4 ± 0.7
45	Orlistat				42.0 ± 2.5

* The inhibition of lipase activity by crude natural extracts was compared to the one observed with the positive control (Orlistat). Data are presented as average ± standard deviation (*n* = 3) and the anti-lipase activity was investigated at a concentration of 100 μg/mL for PPL inhibition.
